# Comment on “Impact of preoperative frailty on venous thromboembolism risk following total hip and knee arthroplasty: a meta-analysis”

**DOI:** 10.1097/MS9.0000000000004335

**Published:** 2025-11-24

**Authors:** Fadong Li, Ning Li

**Affiliations:** School of Clinical Chinese Medicine, Gansu University of Chinese Medicine, Lanzhou, Gansu, China

*To the Editors,*

Lan and colleagues present a timely and comprehensive meta-analysis evaluating the association between preoperative frailty and postoperative venous thromboembolism in patients undergoing total hip or knee arthroplasty^[1]^. The clinical question addressed is highly relevant, and the analytical scope is commendable. We respectfully offer three methodological considerations that may enhance the internal validity and clinical interpretability of the findings. This letter has been prepared in full accordance with the TITAN 2025 Guidelines, ensuring transparent and appropriate use of artificial intelligence tools in academic writing^[2]^.

First, there appears to be a potential unit-of-analysis error. Several included studies contribute multiple correlated effect sizes derived from the same dataset, such as those stratified by procedure type or surgical indication. These effects, however, were treated as independent in the pooled analyses. Such nonindependence may overweight specific data sources, underestimate variance, and narrow confidence intervals, thereby overstating the precision of the summary estimates. Contemporary reporting guidelines, including PRISMA 2020, emphasize the importance of clearly specifying how multiple effects from the same study are handled and of justifying assumptions of independence where applicable^[3]^. We recommend either aggregating within-study cohorts before pooling or applying a three-level random-effects model or robust variance estimation that accounts for clustering at the study level.

Second, the use of common administrative coding to define both exposure and outcome introduces potential measurement bias. In many included cohorts, frailty was assessed using the Hospital Frailty Risk Score, derived from International Classification of Diseases (ICD) codes, while venous thromboembolism was also identified using ICD-based claims data. When both variables originate from the same coding stream, patients with higher coding intensity may appear simultaneously more “frail” and more likely to experience venous thromboembolism, generating artificial correlations driven by documentation rather than underlying biology. Recent work adapting frailty indices from diagnostic codes highlights the importance of validating administrative data when repurposed for clinical phenotyping^[4]^. We encourage prespecified subgroup analyses to distinguish coding-based frailty measures from clinically derived instruments, as well as sensitivity analyses prioritizing imaging-confirmed venous thromboembolism, given current procedure-related event rates and ascertainment practices in arthroplasty populations^[5]^.

Third, the included studies exhibit substantial variability in follow-up duration, ranging from in-hospital to 1-year surveillance, yet the meta-analysis combines odds ratios across these time windows. Longer observation naturally increases the likelihood of event detection and introduces interaction with competing events such as death, which occur more frequently among frail individuals and can bias unadjusted associations. For time-dependent outcomes, effect measures such as the hazard ratio are more appropriate, as is the subdistribution hazard ratio, when nontrivial competing risks are present. Practical guidance recommends either harmonized, fixed follow-up windows or survival analysis methods that explicitly account for competing risks^[6]^. We suggest restricting the primary analysis to a uniform 30-day window, supplemented by time-to-event analyses using cause-specific or Fine–Gray models.

In summary, addressing nonindependence among split datasets, distinguishing coding-derived correlations from true clinical associations, and aligning effect measures with time-at-risk and competing events would enhance both the internal validity and the clinical generalizability of the findings. We offer these observations with the intent of constructively strengthening an already valuable contribution.

## Data Availability

Data sharing does not apply to this article because no new data were generated or analyzed in this study.

## Research registration unique identifying number (UIN)

CRD42024603116.
